# Variability of acquisition phase of computed tomography angiography in acute ischemic stroke in a real-world scenario

**DOI:** 10.1007/s00330-021-08084-5

**Published:** 2021-06-15

**Authors:** Johannes A. R. Pfaff, Bianka Füssel, Marcial E. Harlan, Alexander Hubert, Martin Bendszus

**Affiliations:** 1 Department of Neuroradiology, Heidelberg University Hospital, Im Neuenheimer Feld 400, 69120 Heidelberg, Germany; 2 University Institute for Neuroradiology at Paracelsus Medical University (PMU), University Hospital Salzburg, Christian-Doppler-Klinik, Ignaz-Harrer Straße 79, A-5020 Salzburg, Austria

**Keywords:** Stroke, Computed tomography angiography, Contrast media

## Abstract

**Objectives:**

The informative value of computed tomography angiography (CTA) depends on the contrast phase in the vessels which may differ depending on the level of local expertise.

**Methods:**

We retrospectively measured vessel contrast density from CTA scans in patients presenting with acute ischemic stroke to a comprehensive stroke center (CSC) or to one of eight primary stroke centers (PSC). CTAs were classified into arterial or venous phases as well as into 1 of 5 phases (early arterial, peak arterial, equilibrium, peak venous, and late venous).

**Results:**

Overall, n = 871 CTAs (CSC: n = 431 (49.5%); PSC: n = 440 (50.5%)) were included in the final analysis. A higher venous than arterial contrast density at the level of the circle of Willis was only rarely observed (overall n = 13 (1.5%); CSC: n = 3/431 (0.7%); PCS: n = 10/440 (2.3%); *p* = 0.09). CTAs acquired in the CSC showed more often an early arterial contrast phase (CSC: n = 371 (86.1%); PSC: n = 153 (34.8%), *p* < 0.01). Equilibrium contrast phase, i.e., a slightly stronger arterial contrast with clear venous contrast filling, was more frequent in CTAs from the PSCs (CSC: n = 6 (1.4%); PSC: n = 47 (10.7%); *p* < 0.01).

**Conclusions:**

Despite different technical equipment and examination protocols, the overall number of CTAs with venous contrast was low and did not differ between the CSC and the PCSs. Differences between the further differentiated contrast phases indicate potential for further improvement of CTA acquisition protocols.

**Key Points:**

• *Despite different technical equipment and examination protocols in the diagnostic workup of acute ischemic stroke, the total number of computed tomography angiography (CTA) with venous contrast was low (n = 13/871; 1.5%).*

• *A higher venous than arterial contrast density at the level of the circle of Willis was not more frequent in CTAs from the centers with a high patient volume (comprehensive stroke center) compared to the hospital with lower patient volume (primary stroke centers).*

• *Differences between the further differentiated contrast phases indicate that there is potential for further improvement of CTA acquisition protocols.*

**Supplementary Information:**

The online version contains supplementary material available at 10.1007/s00330-021-08084-5.

## Introduction

Computed tomography angiography (CTA) of the cervical and head vessels is a core element of emergency imaging for stroke. CTA is recommended for the detection of arterial occlusions and stenoses in patients with acute ischemic stroke [1]. In patients with acute hemorrhagic stroke, venous phase of CTA increases spot sign detection, but intracerebral hemorrhage expansion is more likely if a spot sign is detected in arterial phase [2]. Since an arterial contrast phase of the intracranial vessels seems to be desirable in ischemic and hemorrhagic stroke, hospitals often only use one standardized CTA protocol for both. This single-phase protocol may be supplemented with further, later contrast phases [3].

Due to technically different equipment of the hospitals (e.g., CT scanner, contrast agent), individualized examination protocols are used for the acquisition of CTA. Using individualized CTA protocols may lead to a certain variability of the contrast phases of the intracranial vessels in daily practice. However, variability in the contrast phase of a CTA harbors the risk of missed findings or misinterpretations in the diagnosis of vascular occlusions or intracranial collaterals with impact on patient care, as well as reliability and reproducibility of study data.

Our hypothesis is that, because of the common intention to achieve arterial vascular contrast, the variability of the contrast phase in intracranial vessels of CTA examinations carried out in a comprehensive stroke center (CSC) should not significantly differ from the variability of the intracranial contrast phase of CTA examinations that are performed in primary stroke centers (PSC). The purpose of this study was to (1) assess the variability of contrast phases of single-phase CTA and (2), if relevant differences are present, describe the likelihood of acquiring an arterial contrast phase in a real-life scenario.

## Methods

### Study population and data acquisition

The data that support the findings of this study are available from the corresponding author upon reasonable request. This study was approved by the local ethics committee (Ethikkommission der Medizinischen Fakultät Heidelberg No: S-191/2020). Informed consent was waived.

Patients were included in the overall cohort if they received CTA imaging at a university-based comprehensive stroke center or at one of eight primary stroke centers after a neurologist or teleneurologist detected a new acute neurologic deficit within 24 h of symptom onset. Permissible CTAs in the overall cohort were limited to 50% from the comprehensive stroke center and 50% from the primary stroke centers. Every primary stroke center within the teleradiology network contributed at least n = 20 consecutive CTA examinations to the overall cohort. Due to a higher patient volume, consecutive CTAs were performed within a shorter time period in the comprehensive stroke centers, i.e., between January 1, 2019, and April 13, 2019. CTAs at the primary stroke centers were acquired between January 1, 2019, and July 21, 2019.

CTAs from the overall cohort were excluded from entering the final analysis if (1) they showed signs of a delayed or reduced contrasting of the vessels (e.g., due to bilateral preceding stenosis or occlusion, massive contrast reflux into internal jugular vein), (2) they showed signs of a faster or higher contrasting of the venous vessels (e.g., due to arteriovenous shunts by a vascular fistula or malformation), (3) artifacts that prevent reliable measurement of the HU at the level of the circle of Willis, or (4) incomplete retrieval of imaging data from the picture archiving system. If the number of CTA examinations from a primary stroke center fell below 15 as a result of the application of the exclusion criteria, the remaining CTA examinations from the respective primary stroke center were also excluded from the final patient cohort.

### CTA acquisition and image analysis

CTA studies were acquired on commercially available CT scanners. The scanning protocols in the comprehensive stroke center and the teleradiology network were single-phase CTA aiming to begin at the level of the aortic arch and end at the vertex. Image acquisition was triggered by bolus monitoring; imaging parameters are listed in Table 1.

**Table 1:** Computed tomography scanners and CTA acquisition protocols of the hospitals contributing patients to the final analysis

**Hospital**	**CSC**	**PSC1**	**PSC2**	**PSC3**	**PSC4**	**PSC5**	**PSC6**	**PSC7**
**Scanner**	Definition AS 64	Aquilion Prime 80-Slice CT	Emotion 16	Phillips Core 64	Sensation 20	Emotion 16	Sensation 20	Sensation 40
**Manufacturer** **(Sanner)**	Siemens Healthineers, Germany	Canon Medical Systems Corporation, Japan	Siemens Healthineers, Germany	Philips, The Netherlands	Siemens Healthineers, Germany	Siemens Healthineers, Germany	Siemens Healthineers, Germany	Siemens Healthineers, Germany
**Pitch**	1.4	0.813	1.25	1.5	1.2	1.65	1.5	1.3
**Slice thickness CTA source images (mm)**	0.75	0.5	0.6	0.9	0.6	0.5	0.6	0.5
**Contrast agent**	Accupaque 350	Accupaque 350	Optiray 350	Accupaque 300	Accupaque 350	Ultravist 300	Imeron 350	Imeron 350
**Manufacturer** **(contrast agent)**	GE Healthcare Buchler GmbH & Co. KG	GE Healthcare Buchler GmbH & Co. KG	Guerbet	GE Healthcare Buchler GmbH & Co. KG	GE Healthcare Buchler GmbH & Co. KG	Bayer Vital GmbH	Bracco Imaging Deutschland GmbH	Bracco Imaging Deutschland GmbH
**Amount of contrast agent (ml)**	65	80	80	60	65	60	70	60
**Injection rate of contrast agent (ml/s)**	4	4	4	4	4	4.5	4	4
**Amount of saline injected as bolus chaser (ml)**	20	40	50	40	30	30	30	40
**Injection rate of saline (ml/s)**	4	4	4	4	4	4.5	4	4
**Anatomical position of bolus trigger**	Aortic arch	Aortic arch	Aortic arch	Aortic arch	Aortic arch	Aortic arch	Aortic arch	Aortic arch
**Bolus trigger threshold (HU)**	100	180	100	150	62	120	120	115

*CTA* computed tomography angiography, *CSC* comprehensive stroke center, *HU* Hounsfield unit, *PSC* primary stroke center

Two raters (B.F., J.A.R.P.) independently measured intraluminal vessel contrast density (in Hounsfield units, HU) from the CTA scans at predefined anatomical locations (Table 2). Contrast density measurements were obtained by freehand drawn oval region of interest using Centricity™ Universal Viewer (General Electric Company). The size of the region of interests in the corresponding arteries and veins is reported in Supplemental Table 4. Measurements were made on both sides, unless anatomical structures were hypoplastic or unpaired (e.g., superior sagittal sinus, anterior cerebral artery). In order to avoid falsified contrast density values due to limited (collateral) blood flow secondary to a preceding ipsilateral stenosis (i.e., > 70%) or an occlusion [4], measurements in paired structures (e.g., internal carotid artery) were limited to the not affected side. For example, if the right internal carotid artery was occluded, the intracranial contrast phase was classified according to the contrast density measurement made in the left middle cerebral artery only.

**Table 2:** Levels on CTA source images for the measurement of the Hounsfield units at a specific artery and venous structure

Level	Artery	Venous structure
Neck	Common carotid artery (proximal to bifurcation)	Internal jugular vein
Circle of Willis	Proximal M1 segment of the middle cerebral artery	Confluence of sinuses
High convexity	Distal A2 segment of the anterior cerebral artery	Superior sagittal sinus

CTAs were classified into the artery or venous phases (i.e., arterial, when HU in the artery were higher than in the corresponding venous structure; venous, when HU were equal or higher in venous structure) as well as into 1 of the 5 specific phases (early arterial, peak arterial, equilibrium, peak venous, and late venous) based on the methodology described by Rodriguez-Luna et al [2]

In addition, we recorded whether (1) the CTA was started at the level of the ascending aorta above the carina, i.e., thus only comprising the aortic arch, or caudal to it [5], (2) side of contrast injection (right or left antecubital vein), (3) presence of contrast reflux into the internal jugular vein on the site of contrast injection.

### Endpoints

The primary endpoint was the arterial contrast phase of the intracranial vessels at the level of the circle of Willis in CTAs performed at the comprehensive stroke center compared to primary stroke centers. The secondary endpoint was the distribution of contrast phases on the level of the neck, circle of Willis, and high convexity. In a further analysis, the likelihood of the arterial contrast phase of the intracranial vessel at the level of the circle of Willis was investigated.

### Statistical analysis

Statistical analysis was performed by using SPSS Statistics (21.0.0.0; IBM). Continuous variables are presented as means and SD or medians and interquartile intervals, and categorical variables as absolute values and percentages. Differences between groups were assessed with Fisher’s exact test, chi-square test, Mann-Whitney test, or t-test where appropriate, and a two-sided *p*-value of 0.05 was considered statistically significant. Intra- and inter-rater reliability was analyzed using Cohen’s kappa.

Binary logistic regression analysis for the primary endpoint “arterial contrast phase of the intracranial vessels at the level of the circle of Willis” was performed. Since a clear delineation of the cerebral arteries is desirable for the assessment of an intracranial vascular occlusion and the presence of venous contrast, as already prevailing in the equilibrium phase, appears to be disadvantageous, an additional binary logistic regression analysis with the endpoint “sharp arterial contrast,” i.e., comprising only CTAs with early or peak arterial contrast, was performed. Odds ratios (ORs) are described with 95% confidence intervals.

## Results

**Fig. 1:**
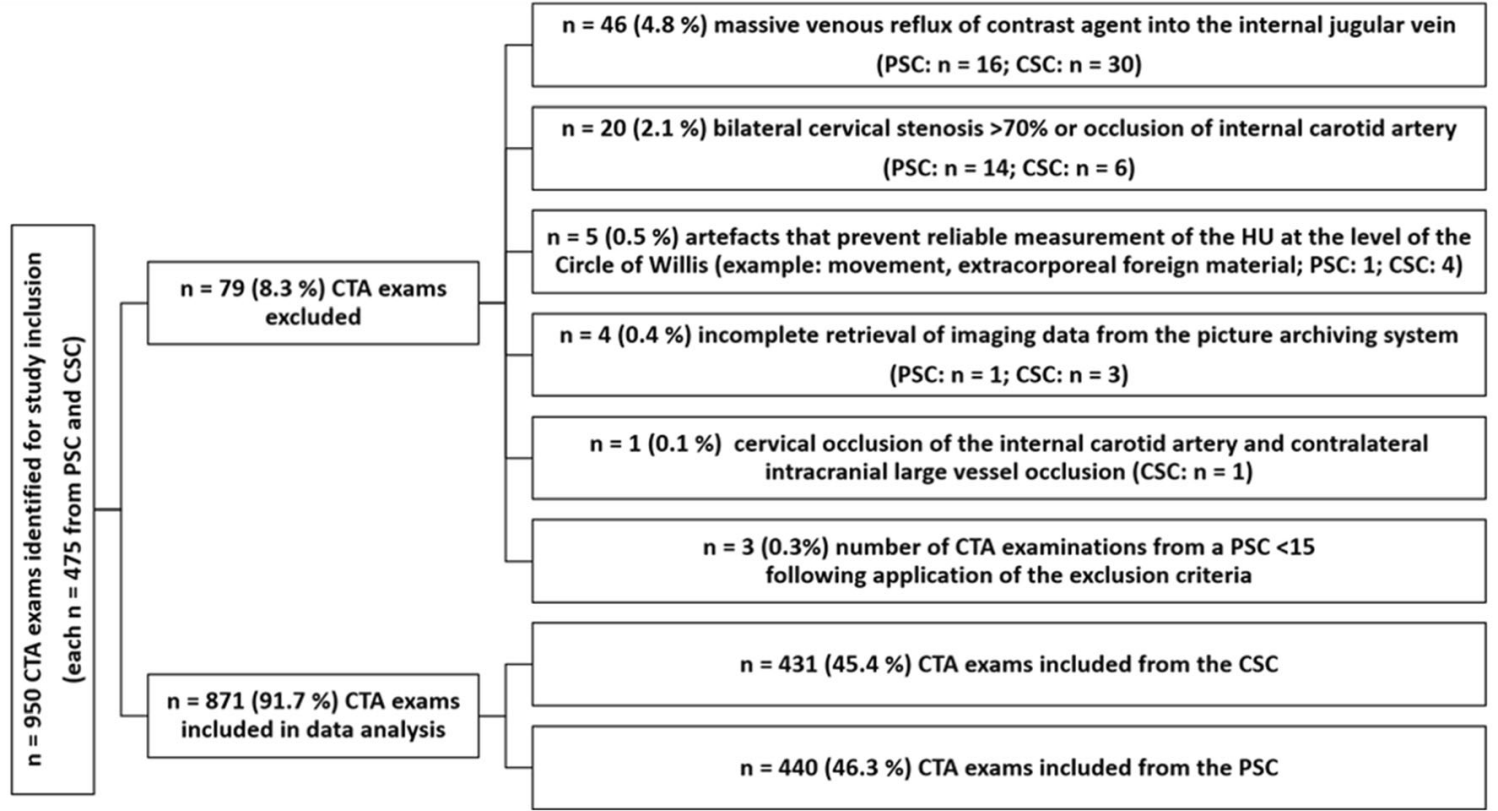
Flowchart of included and excluded CTA exams. CSC = comprehensive stroke center; CTA = computed tomography angiography; PSC = primary stroke center

The overall cohort comprised n = 950 CTAs. Of these, n = 79 patients were excluded (Fig. 1). Thereby, n = 871 CTA, i.e., n = 431 (49.5%) CTA performed at the comprehensive stroke center and n = 440 (50.5%) at seven out of eight primary stroke centers, were included in the final analysis.

Mean patient age was 72 (SD: 14) years, and n = 453 (52.0%) CTAs were performed in female patients. There was no difference regarding age and sex between patients from the comprehensive stroke center and primary stroke centers (Table 3). The injection side of the contrast medium was unknown in n = 43 (4.9%) patients. In the remaining CTA, the contrast injection side was evenly distributed right: n = 395 (45.4%); left: n = 433 (49.7%); *p* = 0.201). CTA covered thoracic structures below the aortic arch in n = 328 (37.7%) patients. This finding was more frequent in patients who received CTA in a primary stroke center (n = 207/440 (47%)) compared to the comprehensive stroke center (n = 121/431 (28.1%); *p* < 0.01). We did not observe any differences between the presence of vascular occlusion (CSC: n = 83/431 (18.7%), PSC: n = 89/440 (20.2%), *p* < 0.734) or the occlusion site (right-sided occlusion: CSC: n = 37/83 (44.6%), PSC: n = 45/89 (50.5%), *p* < 0.269).

**Table 3:** Characteristics of patients and CTA included in the final analysis

	Overall	CSC	PSC	*p*
n (%)	871 (100)	431 (49.5)	440 (50.5)	
Demographic characteristics				
Age, mean (SD), y	72 (14)	72 (15)	73 (14)	.242*
Sex, n (%)				
Female	453 (52)	222 (51.5)	231 (52.5)	.786†
Men	418 (48)	209 (48.5)	209 (47.5)	
CTA characteristics				
Injection side, n (%)				
Right	395 (45.4)	193 (44.8)	202 (45.9)	.201‡
Left	433 (49.7)	222 (51.5)	211 (48.0)	
Unknown	43 (4.9)	16 (3.7)	27 (6.1)	
CTA starting caudal to the aortic arch	328 (37.7)	121 (28.1)	207 (47)	< 0.01†

*Mann-Whitney *U* test; †Fisher’s exact test, two-sided; ‡chi-quadrat nach Pearson, two-sided

*CSC* comprehensive stroke center; *CTA* computed tomography angiography; *PSC* primary stroke center

Regarding allocation of CTAs to either arterial or venous contrast phase according to the contrast density at the level of the circle of Willis, intra-rater reliability was good (kappa: 0.835, [95% CI: 0.613–1], *p* < 0.01) and inter-rater reliability was moderate (kappa: 0.503 [95% CI: 0.018–0.987], *p* = 0.01).

**Fig. 2:**
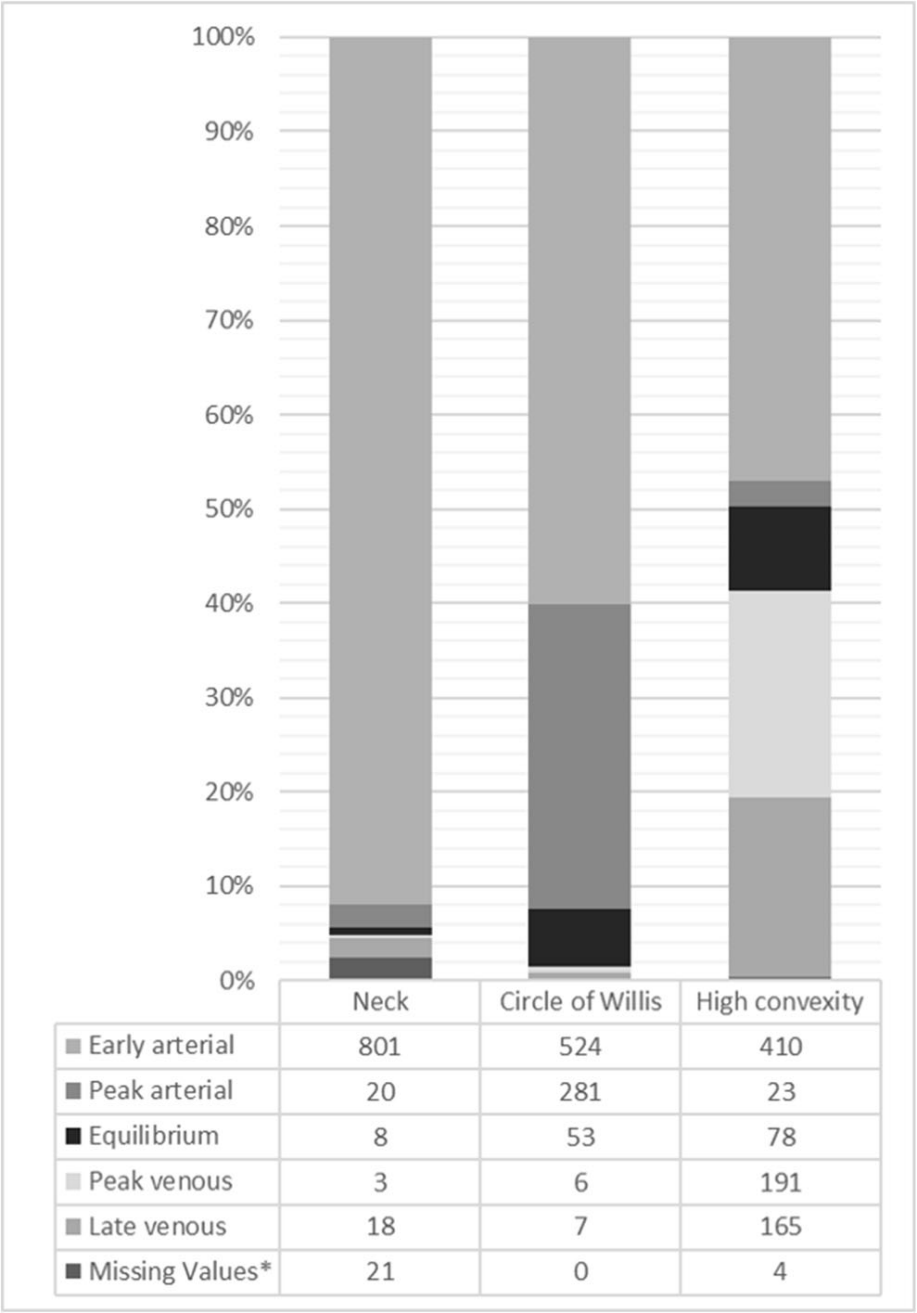
Distribution of CTA studies in each phase according to anatomical level in the final analysis (n = 871). *Missing values due to artifacts (e.g., movement, extracorporeal material) that prevent reliable measurement of the HU. CTA = computed tomography angiography

In the final cohort, a venous contrast phase at the level of the circle of Willis was observed in n = 13/871 (1.5%) CTAs. This finding was not different between CTAs from the comprehensive stroke center compared to primary stroke centers (primary endpoint: CSC: n = 3/431 (0.7%), PSC: n = 10/440 (2.3%); *p* = 0.09, Table 4). The overall number of CTAs with venous contrast phase at the level of the neck and the high convexity was higher (neck: n = 21/850 (2.4%); high convexity: n = 359/867 (40.9%); Fig. 2). In contrast to the finding at the level of the circle of Willis, venously contrasted CTAs at the level of the neck and high convexity were less frequent in CTAs performed at the comprehensive stroke center (neck: *p* < 0.01; high convexity: *p* < 0.01; Table 5).

**Table 4:** Phases of image acquisition by artery and venous structure Hounsfield units according to Rodriguez-Luna et al [2] and number of CTA studies in each phase at the level of the circle of Willis

Phases	Artery	Venous structure	Overall	CSC	PSC
	HU	HU	n (%)	n (%)	n (%)
Early arterial	Higher than venous structure	≤ 200	524 (60.2)	371 (86.1)	153 (34.8)
Peak arterial	≥ 100 higher than venous structure	> 200	281 (32.3)	51 (11.8)	230 (52.3)
Equilibrium	< 100 higher or equal than venous structure	> 200	53 (6.1)	6 (1.4)	47 (10.7)
Peak venous	> 200	Higher than artery	6 (0.7)	1 (0.2)	5 (1.1)
Late venous	≤ 200	Higher than artery	7 (0.8)	2 (0.5)	5 (1.1)

*CSC* comprehensive stroke center; *CTA* computed tomography angiography; *HU* Hounsfield units; *PSC* primary stroke center

**Table 5:** Phases of image acquisition by artery and venous structure Hounsfield units according to Rodriguez-Luna et al [2] and number of CTA studies in each phase at the level of the neck and the high convexity

Anatomical level	Neck			High convexity		
	Overall	CSC	PSC	Overall	CSC	PSC
Phases	n (%)	n (%)	n (%)	n (%)	n (%)	n (%)
Early arterial	801 (92.0)	425 (98.6)	376 (85.5)	410 (47.1)	323 (74.9)	87 (19.8)
Peak arterial	20 (2.3)	1 (0.2)	19 (4.3)	23 (2.6)	5 (1.2)	18 (4.1)
Equilibrium	8 (0.9)	0	8 (1.8)	78 (9.0)	25 (5.8)	53 (12.0)
Peak venous	3 (0.3)	1 (0.2)	2 (0.5)	191 (21.9)	14 (3.2)	177 (40.2)
Late venous	18 (2.1)	2 (0.5)	16 (3.6)	165 (18.9)	63 (14.6)	102 (23.2)
Missing values*	21 (2.4)	2 (0.5)	19 (4.3)	4 (0.5)	1 (0.2)	3 (0.7)

*Missing values due to artifacts (e.g., movement, extracorporeal material) that prevent reliable measurement of density values, i.e., Hounsfield units

*CSC* comprehensive stroke center; *CTA* computed tomography angiography; *HU* Hounsfield units; *PSC* primary stroke center

A differentiated look at the five different contrast phases shows more pronounced alterations. For example, at the level of the circle of Willis, CTAs acquired in the comprehensive stroke center showed more often an early arterial contrast phase, whereas CTAs from primary stroke centers showed more often a peak arterial or equilibrium contrast phase (all *p* < 0.01; Table 4). Similarly, at the level of the neck and the high convexity, arterially contrasted CTAs, especially the early arterial contrasted CTAs, were more frequent in the comprehensive stroke center than in primary stroke centers (all *p* < 0.01, Table 5). Density values of the arteries and veins at different anatomical levels are provided in the data supplement.

In binary logistic regression analysis focusing on the primary outcome parameter, acquisition of CTAs in a comprehensive stroke center was not independently associated with an arterial contrast phase with an OR 3.318 (95% CI 0.907–12.139, *p* = 0.07). When excluding CTAs which showed an equilibrium phase at the level of the circle of Willis, acquisition of CTAs in a comprehensive stroke center was associated with a sharp arterial contrast (OR 6.978 [95% CI 3.409–14.286], *p* < 0.01). Including anatomical structures below the aortic arch in the CTA, was not independently associated with a higher arterial than venous vessel contrast density (OR 1.365 [95% CI 0.417–4.469], *p* < 0.607) or a sharp arterial contrast (OR 0.806 [95% CI 0.485–1.341], *p* < 0.806).

## Discussion

A main result of this retrospective study is that, despite differing, to local settings’ customized CTA exam protocols, only 13 out of 871 (1.5%) CTA in this analysis showed a higher venous than arterial contrast density at the level of the circle of Willis. Furthermore, there was no difference between CTAs from the comprehensive stroke center compared to primary stroke centers. However, in this real-world scenario, CTAs acquired in primary stroke centers tend to show later contrast phases, as can be seen, for example, from a higher number of CTAs with an equilibrium phase at the level of the circle of Willis or predominantly venous contrasting at the high convexity. CTAs that showed an early or peak arterial contrast phase at the level circle of Willis were nearly 7 times more likely to be acquired in the comprehensive stroke center than in the primary stroke centers. Despite a significant difference regarding the thoracic area covered by CTA, starting CTA caudal to the aortic arch does not seem to have had any influence on the contrast phase of the cerebral arteries in our patient cohort.

In patients with acute ischemic stroke, CTA is intended to determine whether a vessel supplying the brain is stenotic or occluded and if so, in accordance with current guidelines, qualifies the patient for further revascularization treatment. In patients with intracranial hemorrhage, CTA is used to identify a spot sign, i.e., extravasation of contrast containing blood, to estimate hemorrhage expansion, or to identify a vascular bleeding source, i.e., aneurysm or arteriovenous malformation. For both reasons, CTA protocols usually aim for a predominantly arterial contrast of the intracranial vessels. Mainly venous contrasting of the intracranial vessels would be undesirable.

In clinical trials, but in particular in hospital networks, imaging evaluation for decision-making for endovascular stroke treatment usually takes place in the center performing endovascular stroke therapy. Therefore, to guarantee a comparable quality of patient care, comparable imaging must be obtained. However, hospitals within a hospital network are most often equipped with different technical and material resources (e.g., CT scanners, contrast agent). To ensure comparability of CTAs, examination protocols are adapted to local conditions. Unfortunately, modifications to exam protocols may lead to a variability in imaging quality.

Based on experience from everyday clinical practice, image assessment on CTA with already beginning venous contrast (including equilibrium phase) may be more prone to errors. This applies, for example, to the differentiation of cortical veins and perpendicular arteries when it comes to the detection of a peripheral vascular occlusion. In acute ischemic stroke patients who received a single-phase CTA, which was evaluated as negative for intracranial occlusion, occult M2 and M3 occlusions could be detected using CT perfusion–based reconstructed angiography [6].

CTA is furthermore used to evaluate clot burden and collaterals. Clot burden and collaterals have been associated with clinical outcome [7]. Different collateral scores were also evaluated as standalone predictors of clinical outcome in patients with acute ischemic stroke [8, 9]. The analysis from Seker et al [8], however, was based on CTA images reconstructed from CT perfusion images from whole brain perfusion. The possibility to select a specific contrast phase or to analyze a time attenuation curve of contrast in a vessel is only possible with perfusion raw data, i.e., repeated image acquisitions that enable a temporal resolution and capture of several contrast phases. Single-phase CTA cannot do this, which is why it is even more important that the single-phase CTA protocol of each hospital must be tailored to obtain the same, desired contrast medium phase in as many applications as possible. This is not only a challenge in everyday clinical practice but is ultimately a quality marker. The contrast phase of a single-phase CTA does, however, not necessarily affect collateral grading or their ability to predict final infarct, if it is arterial or arteriovenous weighted [10].

Computer-aided image evaluation systems can detect intracranial occlusions and categorize the degree of collateral flow on single-phase CTA and thus support the human reader in image evaluation and decision-making [11, 12]. Some of these systems automatically indicate the CTA’s contrast phase of the intracranial vessels [13, 14]. By this, the treating physicians receive an assessment of the quality of the CTA and can thus better assess the result of the computer-aided image evaluation.

In this study, only single-phase CTA examinations were analyzed. Therefore, the generalizability of our results to dual or multiphase CTA is limited. Imaging acquisition for dual- or multiphase CTA consists of two and three imaging acquisitions respectively [3, 15, 16]. In multiphase CTA, the first run is intended to occur during the peak arterial phase, the second during the equilibrium/peak venous phase, and the third run during the late venous phase [3]. Transferring our observations on the variability of the single-phase CTA to these techniques, 7.6% CTAs in our cohort would begin with a delayed phase, i.e., the second or third phase of a multiphase CTA, if a multiphase CTA would have been acquired. Our data cannot answer whether this would have affects image interpretation; however, it appears possible to do so.

There are some limitations due to the retrospective study design, including CTA exams from different hospitals. Another limitation of our study is that possible adjustments to the CTA acquisition of individual patients, for example, a lower flow rate of the contrast medium or the position of the peripheral venous catheter, were not recorded. Likewise, of course, the cardiac output of individual patients could also have influenced the contrast bolus and thus influenced the contrast phase of the intracranial vessels. Non-contrast imaging of the heart might show cardiac enlargement suggestive of a reduced ejection rate and thus could trigger an adjustment of the CTA protocol, but this was not performed in the participating hospitals. Covering anatomic structures below the aortic arch was not uncommon in our patients. Whether this was performed to improve assessments of pulmonary changes (e.g., aspiration pneumonia), pulmonary arteries (in search of a pulmonary embolism), or heart in search of a source of embolism in acute ischemic stroke was not recorded [5, 17, 18].

Furthermore, regarding the different frequency of CTAs in the comprehensive stroke center and in the primary stroke centers, a possible influence of the training of the staff may be present. Nevertheless, the number of CTA examinations with venous contrast at the level of the circle of Willis was low. The study describes the observation from a regional stroke network and thus has limited transferability to other settings.

## **Conclusion**

Despite different technical equipment and examination protocols, the overall number of CTAs with venous contrast was low and did not differ between the comprehensive stroke center and the primary stroke centers. Differences between the further differentiated contrast phases indicate that there is potential for further improvement of CTA acquisition protocols. In large hospital networks or in multicenter studies, the contrast phase in the intracranial arteries should also be considered when evaluating CTA images, assessing image quality, and comparing results.

## Supplementary Information

ESM 1 (DOCX 94 kb)

## Abbreviations

CSC: Comprehensive stroke center
CTA: Computed tomography angiography
HU: Hounsfield unit
PSC: Primary stroke center

## Declarations

### Guarantor

The scientific guarantor of this publication is Dr. Johannes A.R. Pfaff.

### Statistics and Biometry

One of the authors has significant statistical expertise.

### Study subjects or cohorts overlap

Some study subjects or cohorts have been previously reported in Pfaff J, Füssel B, Harlan ME, Hubert A, Bendszus M (2021) Variability of computed tomography angiography coverage of lung parenchyma in acute stroke. Neurol Res Pract. 10.1186/s42466-021-00109-0 (In Press).

### Methodology

• Retrospective

• Case-control study

• Multicenter study
