# Supplementary material for: Variability of acquisition phase of computed tomography angiography in acute ischemic stroke in a real-world scenario
Source: Eur Radiol. 2021 Jun 15;32(1):281–9. doi: 10.1007/s00330-021-08084-5 (PMC8660718; doi:10.1007/s00330-021-08084-5)

# Variability of Acquisition Phase of Computed Tomography Angiography in Acute Ischemic Stroke in a Real-World Scenario.

## Data Supplement (Online)

## Supplementary Tables

Supplemental Table 1 Density values in Hounsfield Units (HU) in the arteries and veins at the level of the neck, the Circle of Willis and the high convexity.

| Anatomical level | | Neck | | | Circle of Willis | | | High Convexity | | |
| --- | --- | --- | --- | --- | --- | --- | --- | --- | --- | --- |
|  |  | Arterial | Venous | Difference | Arterial | Venous | Difference | Arterial | Venous | Difference |
| N | Valid | 850 | 849 | 850 | 871 | 871 | 871 | 868 | 869 | 867 |
|  | Missing  Values* | 21 | 22 | 21 | 0 | 0 | 0 | 3 | 2 | 4 |
|  |  | HU | HU | HU | HU | HU | HU | HU | HU | HU |
| Mean |  | 315,9291 | 83,2204 | 232,8065 | 458,4731 | 190,0246 | 268,4485 | 224,2957 | 203,4367 | 20,9202 |
| SD |  | 302,8250 | 66,0500 | 230,3750 | 449,7500 | 171,5000 | 273,1000 | 213,2500 | 184,8000 | 25,9000 |
| Median |  | 92,21106 | 51,20179 | 98,09010 | 126,25355 | 97,47166 | 122,35497 | 75,70785 | 103,62475 | 93,22479 |
| Minimum |  | 55,20 | 18,70 | -153,40 | 103,50 | 36,70 | -164,40 | 66,00 | 41,50 | -345,50 |
| Maximum |  | 677,00 | 470,45 | 613,50 | 930,25 | 573,50 | 649,20 | 639,00 | 587,80 | 340,80 |
| Percentile | 25 | 256,7250 | 50,3500 | 181,0250 | 378,0000 | 112,0000 | 189,5500 | 170,5625 | 120,8500 | -43,4000 |
|  | 50 | 302,8250 | 66,0500 | 230,3750 | 449,7500 | 171,5000 | 273,1000 | 213,2500 | 184,8000 | 25,9000 |
|  | 75 | 363,9125 | 100,2750 | 284,1000 | 538,3500 | 250,2000 | 350,2500 | 262,0000 | 268,9000 | 85,6500 |

* Missing values due to artefacts (e.g. movement, extracorporeal material) that prevent reliable measurement of density values, i.e. Hounsfield Units.
HU = Hounsfield Units; SD = standard deviation.

Supplemental Table 4 Area in square millimeter of the region of interests in the corresponding arteries and veins on which measurement of density values, i.e. Hounsfield Units, are based.

| Anatomical Level | | Neck | | | | Circle of Willis | | | High Convexity | | |
| --- | --- | --- | --- | --- | --- | --- | --- | --- | --- | --- | --- |
| Anatomical Position | | Common Carotid Artery | | Internal Jugular Vein | | Middle Cerebral Artery | | Confluence of sinuses | Anterior cerebral artery | | Superior Sinus Sagittalis |
|  |  | Right | Left | Right | Left | Right | Left |  | Right | Left |  |
| N | Valid | 848 | 847 | 848 | 846 | 857 | 862 | 871 | 861 | 861 | 869 |
|  | Missing Values* | 23 | 24 | 23 | 25 | 14 | 9 | 0 | 10 | 10 | 2 |
| Mean |  | 4,9134 | 4,6987 | 9,5329 | 8,4853 | 1,2529 | 1,1738 | 4,3890 | 0,4822 | 0,4770 | 2,8641 |
| SD |  | 2,06124 | 2,08301 | 5,75036 | 5,26271 | 0,79326 | 0,72056 | 2,56804 | 0,19876 | 0,24340 | 1,46732 |
| Median |  | 4,6000 | 4,3000 | 8,1000 | 7,2000 | 1,0000 | 0,9300 | 3,7000 | 0,4300 | 0,4300 | 2,7000 |
| Minimum |  | 0,85 | 0,75 | 0,70 | 0,48 | 0,21 | 0,21 | 0,50 | 0,15 | 0,15 | 0,35 |
| Maximum |  | 16,50 | 16,50 | 40,00 | 39,30 | 8,00 | 4,70 | 15,30 | 1,70 | 3,70 | 11,90 |
| Percentile | 25 | 3,4000 | 3,3000 | 5,5000 | 4,9000 | 0,6900 | 0,6375 | 2,5000 | 0,3500 | 0,3300 | 1,8000 |
|  | 50 | 4,6000 | 4,3000 | 8,1000 | 7,2000 | 1,0000 | 0,9300 | 3,7000 | 0,4300 | 0,4300 | 2,7000 |
|  | 75 | 5,9000 | 5,6000 | 12,1000 | 10,4000 | 1,7000 | 1,5000 | 5,5000 | 0,5800 | 0,5600 | 3,6000 |

* Missing values due to artefacts (e.g. movement, extracorporeal material) that prevent reliable measurement of density values, i.e. Hounsfield Units.
SD = standard deviation.

## Supplementary Figures

Supplemental Figure 1 Box plots of the arterial and venous density values (Hounsfield Units) as well as their difference measured at the level of the neck.


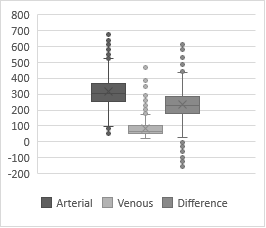


Supplemental Figure 2 Box plots of the arterial and venous density values (Hounsfield Units) as well as their difference measured at the level of the Circle of Willis.


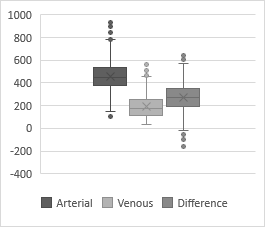


Supplemental Figure 3 Box plots of the arterial and venous density values (Hounsfield Units) as well as their difference measured at the level of the high convexity.


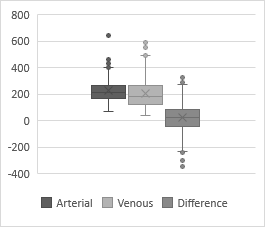

Supplement: Supplementary file 1 — (DOCX 94 kb) [file 330_2021_8084_MOESM1_ESM.docx]
